# Fixed-dose vs loose-dose combination antidiabetic therapy and cardiorenal outcomes in type 2 diabetes: a nationwide comparative effectiveness study

**DOI:** 10.1186/s12933-025-02936-w

**Published:** 2025-09-23

**Authors:** Qiaoling Liu, Paul Welsh, Carlos Celis-Morales, Jennifer S. Lees, Patrick B. Mark, Laura Pazzagli

**Affiliations:** 1 School of Cardiovascular and Metabolic Health, University of Glasgow, Glasgow, UK; 2 Clinical Epidemiology Division, Department of Medicine Solna, Karolinska Institutet, Stockholm, Sweden; 3 Human Performance Lab, Education, Physical Activity and Health Research Unit, Universidad Católica del Maule, Talca, Chile; 4 High-Altitude Medicine Research Centre (CEIMA), Universidad Arturo Prat, Iquique, Chile

**Keywords:** Antidiabetic agent, Comparative effectiveness, Fixed-dose combination, Heart failure, Type 2 diabetes

## Abstract

**Background:**

Combination therapy is gaining attention in type 2 diabetes management due to its potential to reach glycaemic goals within a shorter period. However, the long-term comparative cardiorenal effectiveness of fixed- versus loose-dose combinations remains unclear. This study aimed to assess whether oral antidiabetic fixed-dose combination (FDC) therapy is associated with improved cardiorenal outcomes in adults with type 2 diabetes compared with loose-dose combination (LDC) therapy. A secondary objective was to evaluate the mediating role of medication adherence in these associations.

**Methods:**

This population-based, new-user, active-comparator cohort study used Swedish national registers. Propensity score matching without replacement was applied. Study outcomes included acute myocardial infarction, atrial fibrillation, unstable angina, heart failure, ischaemic stroke, and eGFR < 30 ml/min/1.73m^2^. Associations with cardiorenal outcomes were assessed using Cox regression. Adherence was defined as the proportion of days covered > 80% during the first year.

**Results:**

The median follow-up time was 4.0 years for cardiovascular outcomes and 3.8 years for kidney outcomes. In the matched cohort (mean age 62 years; 67% male), FDC users had higher treatment adherence (68.6 vs. 46.5%). FDC was associated with a lower rate of heart failure (HR = 0.88; 95% CI 0.79, 0.99), with adherence mediating 47% of this association. In people aged ≥ 65 years, FDC was associated with a lower rate of heart failure (HR = 0.79; 95% CI 0.69, 0.91). The observed association was attenuated with further matching for diabetes duration or when drugs were matched at the ATC code level. No associations between FDC use and other outcomes were identified.

**Conclusions:**

FDC therapy in people with type 2 diabetes was associated with a lower rate of heart failure, particularly in older adults. Higher medication adherence appeared to mediate nearly half of this association.

**Supplementary Information:**

The online version contains supplementary material available at 10.1186/s12933-025-02936-w.

## Research in context

**What is currently known about this topic?**

- Guidelines for type 2 diabetes recommend combination therapy for patients at risk of complications.
- Fixed-dose combination drugs are associated with faster attainment of glycaemic goals.
- In patients receiving combination therapy, more than 50% use fixed-dose combination drugs.

**What is the key research question?**

- Is fixed-dose combination drug associated with better cardiorenal outcomes than loose-dose combination drug?

**What is new?**

- In a matched cohort of 27,766 adults, fixed-dose combination drug was associated with a lower heart failure rate.
- Medication adherence mediated 47% of the association.
- In older adults, fixed-dose combination drug was associated with a lower rate of heart failure.

**How might this study influence clinical practice?**

- Findings highlight the need to revisit guidelines to consider formulations in combination therapy.

## Background

The 2024 and 2025 American Diabetes Association (ADA) Standards of Care in Diabetes recommend early combination therapy for type 2 diabetes patients [1, 2]. This approach is preferred over stepwise addition, as it offers faster attainment of glycaemic goals and longer durability of the glycaemic effect [1, 2]. However, while the guidelines promote early combination therapy, they do not specify the optimal formulation, i.e. fixed-dose combination (FDC) versus loose-dose combination (LDC). This study aims to address this research gap by comparing the two formulations.

As a form of combination therapy, FDC refers to combining two or more active ingredients into a single dosage form, while LDC refers to administering the same agents as separate pills. FDC may offer several advantages over LDC, not only by improving medication adherence and reducing the risk of adverse events [3–6], but also by lowering pill burden and simplifying treatment regimens. Additionally, FDC may benefit patients by reducing overall treatment costs and improving dose flexibility, which are important factors influencing treatment satisfaction and long-term outcomes [5, 7, 8] These benefits make FDC a compelling option for long-term diabetes management, especially for patients undergoing polypharmacy. However, LDC may be preferred in certain cases, such as when flexible dose titration is needed or when patients experience side effects from one component of the combination. The choice between FDC and LDC needs to be individualized based on patient characteristics, treatment goals, and tolerability.

Since 2010, the number of approved FDCs has increased, most of which are metformin-based dual-agent combinations [9]. In Sweden, 14 non-insulin oral antidiabetic FDCs are currently available, with 71% (10 out of 14) being metformin-based [10]. Among these, four combine metformin with dipeptidyl peptidase 4 inhibitors (DPP4i), four are metformin with sodium‒glucose cotransporter 2 inhibitors (SGLT2i), and the remaining two combine metformin with thiazolidinedione (TZD).

In Sweden, FDCs account for 4.2% of all prescribed non-insulin antidiabetic drugs filled at pharmacies as of 2024 [11]. Among patients receiving combination therapy, FDC use reaches 37–45% in Japan and 54% in the United States [4, 12].

Current research on FDCs has largely focused on short-term glucose-lowering effects, demonstrating greater reductions in haemoglobin A1c (HbA1c) and fasting plasma glucose [13, 14]. However, the focus of diabetes management has extended from glycaemic control to cardiorenal protection. Hence, the associations of FDCs with long-term cardiorenal outcomes remain unexplored.

The objective of this study is to investigate whether, in adults with type 2 diabetes receiving routine clinical care, FDCs compared with LDCs are associated with lower risks of cardiovascular and kidney outcomes, using a propensity score matched cohort study design.

## Methods

### Study design and population

**Fig. 1:**
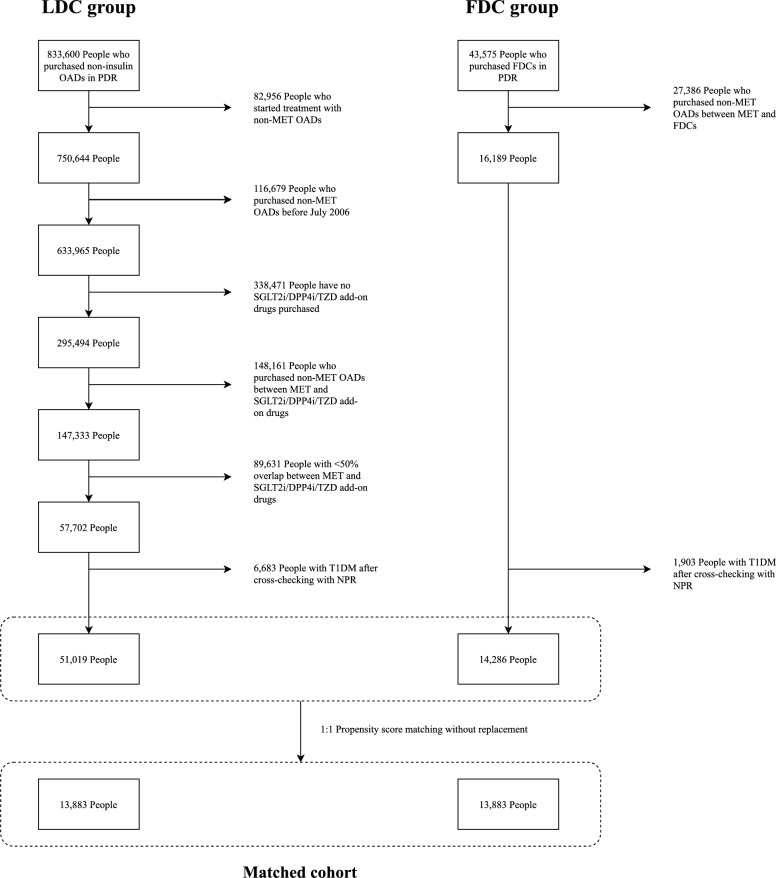
Study population selection flowchart. PDR, Swedish prescribed drug register; MET, Metformin; OAD, Oral antidiabetic drug; SGLT2i, Sodium-glucose cotransporter 2 inhibitor; DPP4i, Dipeptidyl peptidase 4 inhibitor; TZD, Thiazolidinedione; T1DM, Type 1 Diabetes Mellitus; NPR, The Swedish National Patient Register; FDC, Fixed-dose combination; LDC, Loose-dose combination

This study used a new-user active-comparator study design. The study population included individuals with type 2 diabetes who initiated metformin monotherapy between July 1, 2005, and September 30, 2021. Information on filled prescriptions was extracted from the Swedish Prescribed Drug Register (PDR) [15]. The PDR register contains nationwide information on all filled prescriptions in Sweden from July 1, 2005. Prescribed drugs were identified through Anatomical Therapeutic Chemical (ATC) codes (eTable 1). Initiation of metformin therapy was identified by the first dispensed prescription of metformin. The diagnosis of type 2 diabetes was cross-checked using primary diagnosis records from the Swedish National Patient Register (NPR) [16] (Fig. 1).

To ensure a history of metformin monotherapy, a 1-year washout period from the PDR launch was implemented. Patients with medication history of SGLT2i, DPP4i, or TZD within one year prior to the initiation of metformin monotherapy were excluded.

### Comparator groups

The study exposure was the initiation of FDC drugs, defined as the first dispensed prescription of the FDC. FDCs were restricted to combinations of metformin with either an SGLT2i, a DPP4i, or a TZD.

The comparator group of LDCs included individuals receiving metformin and an add-on drug concurrently to emulate the pharmacological exposure of FDC users. Concurrent use was defined as a ≥ 50% overlap time between the dispensingduration of metformin and that of a subsequent add-on drug. The dispensingduration was calculated as the number of packages dispensed multiplied by the number of defined daily dose (DDD) contained in each package. The second agent did not need to be started within a fixed number of days after the purchase of metformin to be considered part of LDC therapy. The relative criterion of 50% provided greater flexibility in identifying LDC therapy. This is because the overlap between metformin and the second agent may occur at the beginning, in the middle, or in the second half of the metformin dispensing duration. A fixed-value criterion would have likely misclassified valid LDC exposed individuals. By applying an intention-to-treat approach, subsequent changes to antidiabetic regimens (additions, discontinuations, or switches) did not alter exposure definition; i.e. follow-up continued regardless.

### Study outcomes

The study outcome was the first incidence of cardiovascular or kidney outcomes during follow-up. The outcomes were selected based on prior studies on SGLT2i/DPP4i/TZD and clinical knowledge [17–22].

The primary outcome included several cardiovascular outcomes, identified from the primary diagnosis in inpatient and specialised outpatient care in the NPR register using the International Classification of Diseases, 10th Revision (ICD-10) code, including unstable angina, myocardial infarction, atrial fibrillation, heart failure, and ischaemic stroke. (eTable 2).

The secondary outcome was kidney impairment, defined as a creatinine-based estimated glomerular filtration rate (eGFRcr) less than 30 ml/min/1.73m^2^. The creatinine-based eGFR was collected from the Swedish National Diabetes Register (NDR) [23]. In NDR, creatinine measurements are collected from patient records during routine clinical visits at specialist clinics and primary care centres. The average interval between creatinine measurements in our study population is 6.7 ± 4.1 months. A lag time of 90 days from the index date was used to avoid reverse causation bias and only events occurring after 90 days were considered. To avoid potential immortal time bias, a follow-up time of at least three months from the index treatment was imposed for all individuals in the study population and person time prior this was not included in the analysis.

### Covariates

A total of 45 baseline covariates were selected, including sociodemographic characteristics [24], comorbidities, and medication history within one year prior to cohort entry, and were extracted from the Total Population Register (TPR) [25], the NPR and the PDR. Additionally, calendar year of cohort entry, cardiovascular or renal events within one year prior to cohort entry [26] and the drug class of the add-on drug/FDC (i.e., SGLT2i/DPP4i/TZD) were included. A detailed list of covariate definitions and data sources can be found in eTable 3. The directed acyclic graph for this study can be found in eFig. 1.

### Calculation of medication adherence

A proxy for medication adherence was assessed using the proportion of days covered (PDC) by medications within 365 days from the index date. Individuals with a PDC greater than 80% were regarded as adherent. The index date was defined as the first day of purchasing the first add-on drug or the FDC. For the FDC group, the numerator was the number of days with the FDC available during the observation period. For the LDC group, the PDC needed to reflect the concurrent use of metformin and the add-on drug; thus, the numerator was the number of days with the add-on drug available during the observation period. Early refills and stockpiling were managed using a carry-over approach, in which overlapping days were shifted forward and the additional supply was appended to the end of the prior dispensing duration. If a refill occurred within the PDC observation window but its dispensing duration extended beyond the window, the coverage was truncated at the end of the window to avoid overestimation of adherence.

### Main analysis

To adjust for confounding and minimise baseline differences between the comparator groups, a 1:1 propensity score matching without replacement was performed, including all covariates. The caliper width was set to 0.20 of the standard deviation of the logit of the propensity score, following published recommendations. [27] Individuals with propensity scores outside the common support region were excluded. A standardized percentage bias of less than 10% after matching was considered a good balance between the two groups.

For the matched cohort, Cox proportional hazards regression models were used to explore the associations between the use of FDC versus LDC and each cardiovascular and renal outcome. Models were fitted with robust standard errors clustered on matched pairs to account for the dependence induced by matching. The proportional-hazards assumption was evaluated using Schoenfeld residuals and no violation was found. Follow-up continued until the earliest occurrence of death, first incidence of the outcome, emigration from Sweden, or end of data availability (September 30, 2022).

### Additional analyses

The mediating role of medication adherence was analysed using the STATA “med4way” package [28] and was presented as the proportion mediated. Stratified analyses were pre-specified for age (≥ 65 years or not), sex (male or female), and drug class (SGLT2i, DPP4i, or TZD), with interaction effects examined.

Additionally, the study population was also stratified by the drug class (SGLT2i/DPP4i/TZD) of the add-on/FDC drugs first, followed by matching and regression analyses within each stratum.

For sensitivity analyses, first, the cohort was matched on the duration of type 2 diabetes by cohort entry (obtained from the NDR) in addition to all covariates. The duration of diabetes was defined as the time interval between the first recorded diagnosis of type 2 diabetes and the index date of the antidiabetic treatment. Second, the cohort was matched on paired ATC codes instead of the drug class. Specifically, an FDC (e.g., A10BD15: metformin and dapagliflozin) was paired with its corresponding LDC (e.g., A10BK01: dapagliflozin) (eTable 4). Third, to assess the robustness of the results to residual immortal time bias, the lag time was removed and all the events occurring during the 90-day lag time were included as outcomes, and the primary analysis was repeated. Fourth, the primary analysis was repeated in a subset population without history of chronic ischaemic heart disease, myocardial infarction, and unstable angina.

## Results

### Baseline population characteristics

In the unmatched population, the LDC group had 51,019 people, and the FDC group had 14,286 people. Individuals with a filled prescription for SGLT2i, whether in FDC or LDC form, had a higher prevalence of angina, ischaemic heart disease, myocardial infarction, and recent CVD events (13 ~ 14%) compared to other drug classes (3 ~ 7%) (eTable 5). Each year, approximately 80% of the study population was in the LDC group (eTable 6). The use of SGLT2i has increased over time, and the proportion of DPP4i usage has initially increased but has slightly declined in recent years, whereas the use of TZD drugs has consistently decreased and has become less common in recent years (eTable 7).

After 1:1 propensity score matching without replacement, both groups included 13,883 individuals, with a total matched cohort of 27,766 people. The mean ± standard deviation (SD) of age was 61.8 ± 11.9 years in the LDC group and 62.0 ± 11.7 years in the FDC group. The proportion of males was 67.0% in both groups (Table 1).

**Table 1:** Unmatched and matched major baseline characteristics of the study population

	Unmatched population		Matched population		Standardized
	LDC	FDC	LDC	FDC	Percentage bias
N (row%)	51,019 (78.1)	14,286 (21.9)	13,883 (50.0)	13,883 (50.0)	NA
Age in year, mean (SD)	63.1 (11.8)	61.9 (11.8)	61.8 (11.9)	62.0 (11.7)	2.0
Male, N (%)	32,854 (64.4)	9570 (67.0)	9295 (67.0)	9296 (67.0)	0.0
PDC, median (IQR)	0.70 (0.49, 1.00)	0.98 (0.70, 1.00)	0.72 (0.47, 1.00)	0.98 (0.71, 1.00)	NA
Adherence, N (%)	23,417 (45.9)	9750 (68.3)	6452 (46.5)	9519 (68.6)	NA
Antidiabetic drug class, N (%)					
SGLT2i	20,136 (39.5)	5625 (39.4)	4722 (34.0)	5583 (40.2)	12.7
DPP4i	28,537 (55.9)	7191 (50.3)	7724 (55.6)	6863 (49.4)	− 12.4
TZD	2346 (4.6)	1470 (10.3)	1437 (10.4)	1437 (10.4)	0.0
Comorbidities, N (%)					
Atrial fibrillation	3599 (7.1)	819 (5.7)	774 (5.6)	815 (5.9)	1.2
Angina	5047 (9.9)	1449 (10.1)	1383 (10.0)	1441 (10.4)	1.4
Atherosclerosis	631 (1.2)	156 (1.1)	157 (1.1)	156 (1.1)	− 0.1
Cardiomyopathy	438 (0.9)	108 (0.8)	102 (0.7)	106 (0.8)	0.3
Heart failure	2044 (4.0)	439 (3.1)	376 (2.7)	436 (3.1)	2.3
Ischaemic heart disease	4799 (9.4)	1448 (10.1)	1320 (9.5)	1441 (10.4)	2.9
Myocardial infarction	4708 (9.2)	1284 (9.0)	1159 (8.3)	1278 (9.2)	3.0
Ischaemic stroke	2323 (4.6)	530 (3.7)	491 (3.5)	529 (3.8)	1.4
Haemorrhage stroke	530 (1.0)	115 (0.8)	110 (0.8)	115 (0.8)	0.4
Hypertension	4803 (9.4)	1213 (8.5)	1217 (8.8)	1207 (8.7)	− 0.3
Acute kidney injury	208 (0.4)	41 (0.3)	34 (0.2)	41 (0.3)	0.9
CKD Stage 1	23 (< 0.1)	5 (< 0.1)	5 (< 0.1)	5 (< 0.1)	0.0
CKD Stage 2	43 (0.1)	10 (0.1)	15 (0.1)	10 (0.1)	− 1.3
CKD Stage 3	85 (0.2)	12 (0.1)	11 (0.1)	12 (0.1)	0.2
Proteinuria	56 (0.1)	< 5 (< 0.1)	< 5 (< 0.1)	< 5 (< 0.1)	0.0
Diabetic nephrology	140 (0.3)	26 (0.2)	26 (0.2)	26 (0.2)	0.0
Diabetic neuropathy	215 (0.4)	55 (0.4)	55 (0.4)	55 (0.4)	0.0
Diabetic retinopathy	1983 (3.9)	497 (3.5)	488 (3.5)	496 (3.6)	0.3
Anaemia	734 (1.4)	159 (1.1)	131 (0.9)	159 (1.1)	1.8
Alcoholic liver disease	102 (0.2)	23 (0.2)	22 (0.2)	23 (0.2)	0.2
Cirrhosis	94 (0.2)	36 (0.3)	35 (0.3)	35 (0.3)	0.0
Chronic hepatitis	443 (0.9)	104 (0.7)	101 (0.7)	101 (0.7)	0.0
Hyperthyroidism	703 (1.4)	158 (1.1)	142 (1.0)	157 (1.1)	1.0
Hypoparathyroidism	265 (0.5)	59 (0.4)	51 (0.4)	59 (0.4)	0.8
Asthma	1280 (2.5)	332 (2.3)	340 (2.4)	330 (2.4)	− 0.5
Smoking	86 (0.2)	22 (0.2)	18 (0.1)	22 (0.2)	0.7
Drinking	1241 (2.4)	292 (2.0)	290 (2.1)	291 (2.1)	0.0
Dyslipidemia	557 (1.1)	234 (1.6)	225 (1.6)	234 (1.7)	0.6
Obesity	1552 (3.0)	441 (3.1)	446 (3.2)	438 (3.2)	− 0.3
Recent CVD events^a^, N (%)	4659 (9.1)	1124 (7.9)	1016 (7.3)	1114 (8.0)	2.5
Recent renal events^b^, N (%)	186 (0.4)	28 (0.2)	34 (0.2)	28 (0.2)	− 0.8
Drug, N (%)					
Antihypertensives	35,474 (69.5)	9642 (67.5)	9343 (67.3)	9482 (68.3)	2.2
Beta blockers	23,856 (46.8)	6436 (45.1)	6224 (44.8)	6353 (45.8)	1.9
Calcium channel blocker	20,557 (40.3)	5464 (38.2)	5326 (38.4)	5396 (38.9)	1.0
Diuretics	17,748 (34.8)	4496 (31.5)	4382 (31.6)	4454 (32.1)	1.1
Lipid regulators	38,087 (74.7)	10,247 (71.7)	9860 (71.0)	10,067 (72.5)	3.4
Antithrombotics	24,571 (48.2)	6645 (46.5)	6399 (46.1)	6532 (47.1)	1.9
Corticosteroids	13,150 (25.8)	3503 (24.5)	3458 (24.9)	3479 (25.1)	0.3
NSAIDs	34,176 (67.0)	9120 (63.8)	8994 (64.8)	9037 (65.1)	0.7
Marital status					
Unmarried	10,968 (21.7)	2863 (20.6)	2875 (20.7)	2863 (20.6)	− 0.2
Married	26,069 (51.6)	7617 (54.8)	7632 (55.0)	7613 (54.8)	− 0.3
Separated	9619 (19.0)	2533 (18.2)	2522 (18.2)	2533 (18.2)	0.2
Widowed	3864 (7.6)	874 (6.3)	854 (6.2)	874 (6.3)	0.6

SGLT2i, Sodium-glucose cotransporter 2 inhibitor; DPP4i, Dipeptidyl peptidase 4 inhibitor; TZD, Thiazolidinedione; CKD, Chronic kidney disease; CVD, Cardiovascular disease; NSAID, Nonsteroidal anti-inflammatory drug; LDC, loose-dose combination; FDC, Fixed-dose combination; PDC, Proportion of days covered; IQR, Interquartile range

Numbers smaller than five were presented as “ < 5” in accordance with data anonymization standards to minimize the risk of re-identification

^a^Recent CVD events: Angina, atherosclerosis, atrial fibrillation, cardiomyopathy, chronic ischaemic heart disease, heart failure, myocardial infarction, and stroke, occurring within 1 year prior to the cohort entry

^b^Recent kidney events: Acute kidney injury, CKD Stage 1 ~ 3, and proteinuria occurring within 1 year prior to the cohort entry

Matched baseline characteristics were well balanced for most covariates, with a standardized percentage of bias between − 10.0 and 10.0% (e.g., age: 2.0%, sex: 0.0%, comorbidities: − 1.3–3.0%, current medication: 0.3–3.4%). Minor imbalances persisted in the distribution of antidiabetic drug classes and geographic regions (Table 1, eTable 8). No meaningful imbalances were observed for key demographic or clinical characteristics.

Angina was the most prevalent comorbidity in the matched groups (10.0% FDC and 10.4% LDC), followed by ischaemic heart disease (9.5 and 10.4%, respectively). The use of lipid-lowering agents was found in more than 70% of the people in both groups, whereas more than 60% of the people in both groups used antihypertensive drugs or non-steroidal anti-inflammatory drugs. The FDC group showed higher PDC (median [IQR]: 0.98 [0.71, 1.00] vs. 0.72 [0.47, 1.00]) and better medication adherence (68.6 vs. 46.5%) than the LDC group during the first year after the index treatment (Table 1).

### Cardiovascular outcome analyses

In both the unmatched and matched populations, heart failure was the most common cardiovascular outcome, followed by unstable angina and acute myocardial infarction. In the matched population, the proportion of outcomes was generally similar between groups, except for heart failure, which was more common in the LDC group (eTable 9). Heart failure had the highest incidence rate of 8.7 cases per 1,000 person-years (95% Confidence Interval [CI]: 8.2, 9.2), while unstable angina had the lowest, 2.8 cases per 1,000 person-years (95% CI 2.5, 3.0) (Table 2).

**Table 2:** Hazard ratios in the overall population after propensity score matching

	Acute MI	Atrial fibrillation	Unstable angina	Heart failure	Ischaemic stroke	eGFRcr < 30 ml/min/1.73m^2^
Events during follow-up, N	934	732	404	1250	732	185
Follow-up time in years, Median (IQR)	4.0 (1.9, 7.3)	4.0 (1.9, 7.3)	4.0 (1.9, 7.4)	4.0 (1.9, 7.3)	4.0 (1.9, 7.3)	3.8 (1.9, 6.3)
Incident rate per 1000 person-years, (95%CI)						
Overall	6.5 (6.1, 6.9)	5.0 (4.7, 5.4)	2.8 (2.5, 3.0)	8.7 (8.2, 9.2)	5.0 (4.7, 5.4)	2.8 (2.5, 3.3)
LDC	6.6 (6.0, 7.2)	5.1 (4.6, 5.6)	2.9 (2.5, 3.3)	9.2 (8.5, 9.9)	4.9 (4.4, 5.4)	3.0 (2.5, 3.7)
FDC	6.4 (5.8, 7.0)	5.0 (4.5, 5.5)	2.7 (2.3, 3.1)	8.1 (7.5, 8.8)	5.2 (4.7, 5.8)	2.6 (2.1, 3.3)
Hazard Ratio (95%CI)	0.98 (0.86, 1.11)	0.98 (0.85, 1.14)	0.93 (0.77, 1.13)	0.88 (0.79, 0.99)	1.07 (0.92, 1.24)	0.89 (0.67, 1.19)

Sample size for cardiovascular outcomes is 27,766, for kidney outcome, the size is 13,728

MI, Myocardial infarction; eGFR, Estimated glomerular filtration rate

**Fig. 2:**
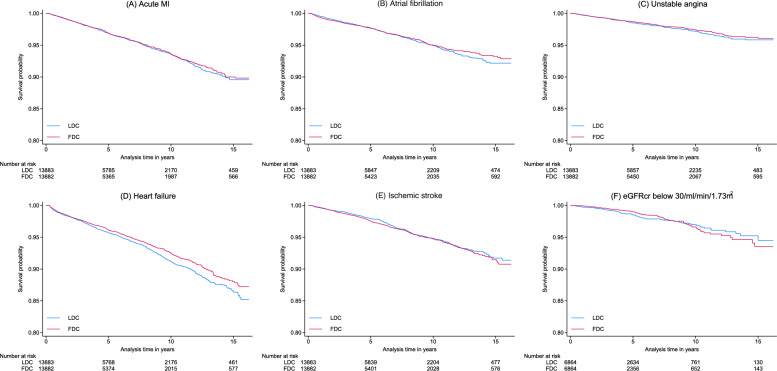
Kaplan-Maier survival curves for outcomes. **A** Acute MI; **B** Atrial fibrillation; **C** Unstable angina; **D** Heart failure; **E** Ischaemic stroke; **F** eGFRcr below 30 ml/min/1.73m^2^. FDC, fixed-dose combination; LDC, loose-dose combination; MI, myocardial infarction; eGFR, estimated glomerular filtration rate

In the matched cohort, the median follow-up duration was four years for heart failure and was highly consistent across all other cardiovascular outcomes. A total of 1250 heart failure cases were observed during follow-up. The incidence rate of heart failure was lower in the FDC group (8.1 vs. 9.2 per 1000 person-years) (Table 2). The FDC group had a lower rate of heart failure (Hazard Ratio [HR] = 0.88; 95% CI 0.79, 0.99). Medication adherence mediated 47% (95% CI 5, 90%) of this association. The absolute risk differences in heart failure between FDC and LDC users aged 65 years or above at 3, 5, and 10 years are − 1.0, − 1.6, and − 3.1%, respectively. When assessing the mediating effect of continuous PDC on the association between FDC and heart failure incidence, the result was consistent, with continuous PDC mediating 48% (95% CI 0, 97%) of the association. The Kaplan‒Meier survival graph showed paralleled curves between groups. No differences were observed in other cardiovascular outcomes (Table 2, Fig. 2A–E).

In the stratified analysis, in people aged 65 years and older, FDC was associated with a lower rate of heart failure (HR = 0.79; 95% CI 0.69, 0.91), with evidence of interaction effect (*P* = 0.03). There was no evidence of a sex interaction (*P* = 0.73). Female FDC initiators also had a lower rate of acute myocardial infarction (HR = 0.78; 95% CI 0.60, 1.00) (Table 3).

**Table 3:** Hazard ratios for the stratified analyses after propensity score matching

	Sample size	Acute MI	Atrial fibrillation	Unstable angina	Heart failure	Ischaemic stroke	Sample size^a^	eGFRcr < 30 ml/min/1.73m^2a^
Hazard Ratio (95%CI)								
Age								
Below 65 years	15,668	1.06 (0.88, 1.28)	1.01 (0.80, 1.29)	1.02 (0.77, 1.34)	1.03 (0.86, 1.25)	1.11 (0.88, 1.39)	7321	1.37 (0.76, 2.44)
65 years and older	12,098	0.89 (0.74, 1.06)	0.95 (0.79, 1.14)	0.84 (0.64, 1.11)	0.79 (0.69, 0.91)	1.02 (0.85, 1.23)	6407	0.74 (0.53, 1.04)
Sex								
Male	18,591	1.06 (0.91, 1.23)	1.02 (0.86, 1.21)	0.97 (0.78, 1.21)	0.90 (0.78, 1.02)	1.15 (0.96, 1.37)	9195	0.89 (0.59, 1.33)
Female	9175	0.78 (0.60, 1.00)	0.90 (0.68, 1.18)	0.81 (0.53, 1.24)	0.86 (0.70, 1.06)	0.92 (0.71, 1.19)	4533	0.89 (0.59, 1.36)
LDC/FDC Drug class								
SGLT2i	10,305	1.10 (0.80, 1.50)	1.20 (0.88, 1.63)	1.18 (0.78, 1.77)	0.87 (0.69, 1.10)	1.12 (0.75, 1.66)	5622	5.17 (0.60, 44.60)
DPP4i	14,587	0.95 (0.80, 1.13)	0.92 (0.76, 1.11)	0.91 (0.70, 1.18)	0.87 (0.75, 1.01)	1.04 (0.86, 1.25)	7341	0.85 (0.60, 1.19)
TZD	2874	0.91 (0.71, 1.18)	0.91 (0.63, 1.30)	0.76 (0.49, 1.16)	0.89 (0.70, 1.13)	1.08 (0.82, 1.42)	765	0.90 (0.48, 1.67)

SGLT2i, Sodium-glucose cotransporter 2 inhibitor; DPP4i, Dipeptidyl peptidase 4 inhibitor; TZD, Thiazolidinedione; MI, Myocardial infarction; eGFR, Estimated glomerular filtration rate; FDC, Fixed-dose combination; LDC, Loose-dose combination

^a^Sample size of this column is for the outcome eGFRcr < 30 ml/min/1.73m^2^ only

When stratified by drug class after matching, FDC users of metformin plus DPP4i did not have a significantly different risk of heart failure compared to LDC users, with a CI crossing the null (HR = 0.87; 95% CI 0.75, 1.01). No differences were observed in other cardiovascular outcomes (Table 3).

A summary table of sensitivity analyses can be found in the supplementary material (eTable 11). Additional matching on diabetes duration reduced the study population by 34.3%, owing to the reduced population coverage of the NDR (the source of duration data), leaving 18,250 individuals. The average duration of type 2 diabetes was 6.6 ± 5.4 and 6.6 ± 5.6 years in the LDC and FDC groups, respectively. No differences were observed in any CVD outcomes. (eTable 12).

After matching at ATC level, the matched population was as well balanced as the population matched at the drug class level, with minor imbalances remaining in three counties. The association of FDC use with heart failure was not significantly lower in the unstratified population (HR = 0.91; 95% CI 0.80, 1.02) (eTable 13). Stratified analyses on heart failure outcomes showed that individuals aged 65 years and older had a lower rate of heart failure (HR = 0.82; 95% CI 0.71, 0.94) when initiating FDCs (eTable 14). The results were consistent using outcomes occurring at any time after the index date (eTable 15), and in people without history of chronic ischaemic heart disease, myocardial infarction, and unstable angina (eTables 16, 17).

### Kidney outcome analysis

In the unmatched 65,305 people, 33,849 people (51.8%) did not have creatinine data available. The missing rate was similar in LDC and FDC groups (52.7 and 47.3% respectively). In the matched population of 13,728 people, the mean baseline eGFRcr was 91 ± 25 and 91 ± 24 ml/min/1.73m^2^ in the LDC and FDC groups, respectively. With a median follow-up of 3.8 years (95% CI 1.9, 6.3), the incidence rate was 2.8 cases per 1000 person-years (95% CI 2.5, 3.3). A total of 185 cases was identified, with 83 in the FDC group and 102 in the LDC group. No associations between FDC and kidney events were observed. (Fig. 2F, Table 3, eTables 10, 12, 13, 15).

## Discussion

In this real-world cohort study of people with type 2 diabetes who initiated treatment with metformin, the use of FDCs was associated with a modestly lower rate of heart failure than the LDC regimen was, and 47% of this was mediated by medication adherence. This association was also observed in people aged 65 years and older, but was attenuated with further matching for diabetes duration or when drugs were matched at ATC code level instead of drug classes. No associations were observed for myocardial infarction, atrial fibrillation, unstable angina, stroke, or kidney impairment.

The observed cardiovascular protection of FDC therapy on heart failure could be partly explained by differences in medication adherence, since poor adherence could lead to an increased risk of CVD events [29]. The FDC initiators in this study had higher adherence. FDCs reduce pill burden and simplify treatment, which may improve patients’ consistency in taking medications as prescribed.

The larger association of FDCs with heart failure in people aged 65 years and older, could be explained by age-related alterations in pharmacokinetics and pharmacodynamics [30]. Older adults more frequently experience declined kidney function, leading to reduced drug clearance and prolonged plasma drug concentrations. Additionally, the higher prevalence of multimorbidity and polypharmacy in older adults may further influence drug absorption and excretion, potentially leading to increased drug exposure when taking FDCs.

Analysis stratified on drug class suggested that FDCs of metformin with DPP4i may be associated with a lower rate of heart failure. One trial has shown metformin and sitagliptin combination associated with improved glucose control compared to metformin monotherapy, which may indirectly reduce the risk of complications [31]. However, another study reported that the addition of saxagliptin to existing antidiabetic therapy was associated with an increased risk of hospitalization for heart failure (HR = 1.27; 95% CI 1.07, 1.51) [32]. Notably, that study did not include a direct comparison with metformin monotherapy, although metformin accounted for approximately 70% of concomitant antidiabetic use throughout the trial. Another important concern is around the 40% concomitant use of sulfonylureas, as sulfonylureas have been associated with elevated cardiovascular risk. Finally, saxagliptin in that study was not administered as an FDC. Therefore, it was an LDC-versus-LDC study. Consequently, the study provides only limited direct inference for our findings.

In analyses stratified on drug class, the observed differences may be attributable to factors inherent to the FDC itself. FDCs are designed to control drug release kinetics and enhance the physical and chemical stability of each component within the gastrointestinal environment. FDCs often incorporate specialized excipients or matrix systems designed to prevent the degradation of labile compounds in the presence of others, a limitation seen in LDCs [33]. This approach is useful, as several antidiabetic agents in this study, such as empagliflozin and saxagliptin, are labile under hydrolytic or oxidative stress conditions [34, 35]. Additionally, co-granulation, modified release layers, or enteric coatings can yield measurable differences in bioavailability and therapeutic performance. However, since our study did not include pharmacokinetic or bioavailability data, formulation-related mechanisms should be considered speculative in explaining our findings. Finally, in additional analyses in populations with stratification by drug classes pre-matching, no associations were observed.

There were no differences in acute myocardial infarction, ischaemic stroke, unstable angina, or atrial fibrillation between the FDC and LDC treatment groups. To date, only a few SGLT2i (e.g., canagliflozin) have been recommended by regulatory agencies to reduce the risk of major adverse cardiovascular events in people with known atherosclerotic CVD, but not in people at risk of those diseases [36]. In terms of cardiovascular events, the major protective effect of SGLT2i observed is on heart failure [36]. However, published research has not shown evidence of beneficial cardiovascular effects of DPP4i [37].

As yet, SGLT2i has shown kidney-protective effects, but there is insufficient evidence supporting the use of DPP4i [37]. This study has observed no associations between FDC and the kidney outcome (eGFRcr < 30 ml/min/1.73m^2^). This may be due to the suboptimal capture of kidney events, the short follow-up duration (median: 3.8 years), and the generally healthy status of the kidneys at baseline in both groups, resulting in low power. Because only 185 renal events accrued, statistical power was limited. Using Schoenfeld’s approximation (two-sided α = 0.05, 1:1 allocation), with 185 events the minimum detectable effect at 80% power was around HR = 0.66. Thus, the null finding for the renal outcome likely reflects limited power and not necessarily absence of effect. Further studies on people with impaired kidney function and longer follow-up periods are encouraged.

Despite stratification and matching, baseline cardiovascular disease differed across drug classes, especially among SGLT2i users, indicating prescribing bias by cardiovascular risk. This may cause confounding by indications, potentially masking the true benefits of FDC in high-risk patients, which could explain part of the attenuation in associations observed in the analyses stratified by drug class and with matching at ATC level.

This study contributes to the clinical management of type 2 diabetes by examining the role of FDCs in combination therapy, thereby clarifying a previously ambiguous area within current guidelines. To our knowledge, this is the first study that has specifically examined cardiorenal endpoints for FDC versus LDC therapy in type 2 diabetes using real-world data.

This study has several strengths, such as the large, real-world cohort drawn from routine clinical practice, which enhances the generalizability of the findings to broader populations. Additionally, the use of an active comparator provides a rigorous reference group, and a wide range of baseline characteristics and comorbidities were adjusted for to minimize confounding.

Despite these strengths, several limitations must be acknowledged. First, the exposure definition relied on prescription fill records, which do not provide information on the actual duration and doses. Second, there were imbalances in drug classes after matching, which could have biased the results of the main analysis. However, stratified analysis on each drug class showed possible negative associations with heart failure in DPP4i FDC users. Third, the sensitivity analysis using matching at ATC code level showed similar trends as those of the primary findings but an attenuation in associations. While matching at the ATC code level may offer a greater control over potential confounding by indication, matching at the drug class level is more aligned with the original aim of the study to compare the form of combination therapies (FDC versus LDC), independently from individual drug effect. Additionally, current clinical guidelines (1) recommend combination therapies based on drug classes (e.g., metformin in combination with SGLT2i) rather than specific drugs (e.g., dapagliflozin). Therefore, drug class-level matching enhances the clinical generalizability of our findings. Fourth, the proportion of days covered is a proxy for adherence, and this is only an approximation of true medication-taking behaviour [38]. Fifth, the follow-up time, although sufficient to observe differences in heart failure, may not have been long enough to detect divergences in outcomes such as chronic kidney disease progression or hard atherosclerotic events, which develop over many years. Sixth, the intention-to-treat approach did not reflect the drug-switching during the follow-up duration. In the matched population, 25.6% of FDC users switched to LDC or non-metformin monotherapy, compared to 8.5% of LDC users. There were also users who switched to a different treatment group and later returned to their original treatment. This imbalance in switching may dilute the differences between FDC and LDC therapies, potentially underestimating the true comparative effectiveness of FDC. However, the mediation analysis has partially offset this disadvantage, showing that being adherent is an important mediator. Seventh, GLP-1 receptor agonists were not included in this study because no FDCs containing GLP-1 RAs were available on the market at the time of data collection. Future research on GLP-1 RAs is required. Eighth, potential informative censoring like death and emigration may bias the results. However, the direction of the effect is unknown. All-cause mortality during follow-up was 8.0% in FDC users and 8.6% in LDC users. The emigration rate was 0.94% and 0.68% for FDC and LDC users respectively. Given these small and similar rates, any differential informative censoring is expected to be modest and unlikely to explain the results. Finally, this study was conducted within a specific healthcare system. Differences in FDC availability, prescribing practices, and government subsidies may limit the generalizability of the findings to other countries.

These findings suggest that the use of FDCs is associated with a lower rate of heart failure in people with type 2 diabetes, but not with a reduced risk of other cardiovascular outcomes. The association is partially mediated by improved adherence. This strategy could be particularly relevant for older adults or those facing polypharmacy. In health care systems with low FDC uptake, FDCs may serve as a practical tool to simplify treatment, improve adherence, and reduce heart-failure burden. Priority should be given to clinically equivalent FDCs for adults with type 2 diabetes who require dual oral therapy, especially older patients and those on polypharmacy, with parity (or better) reimbursement to reduce access barriers. Due to the neutral results for non-heart failure outcomes, policy implementation should proceed via evidence-guided pilots with prospective monitoring. While the results highlight a potential clinical advantage of FDC use, especially in routine clinical care settings, further studies are encouraged to focus on pragmatic trial designs, long-term kidney outcomes, and high-risk groups such as patients with impaired kidney function.

## Supplementary Information

Additional file1 (DOCX 205 KB)

## Data Availability

Personal data used in this study are protected by privacy laws in Sweden, and therefore, are not publicly available.

## Abbreviations

ADA: American Diabetes Association
ATC: Anatomical therapeutic chemical
CVD: Cardiovascular
DDD: Defined daily dose
DPP4i: Dipeptidyl peptidase 4 inhibitors
eGFR: Estimated glomerular filtration rate
FDC: Fixed-dose combination
ICD-10: International classification of diseases, 10th Revision
LDC: Loose-dose combination
NDR: Swedish national diabetes register
NPR: Swedish national patient register
PDC: Proportion of days covered
PDR: Swedish prescribed drug register
SGLT2i: Sodium-glucose cotransporter 2 inhibitors
TPR: Total population register
TZD: Thiazolidinedione
