# Supplementary material for: Fixed-dose vs loose-dose combination antidiabetic therapy and cardiorenal outcomes in type 2 diabetes: a nationwide comparative effectiveness study
Source: Cardiovasc Diabetol. 2025 Sep 23;24:365. doi: 10.1186/s12933-025-02936-w (PMC12455815; doi:10.1186/s12933-025-02936-w)
Supplement: Supplementary file 1 — Additional file1 (DOCX 205 KB) [file 12933_2025_2936_MOESM1_ESM.docx]

**Fixed-dose vs loose-dose combination antidiabetic therapy and cardiorenal outcomes in type 2 diabetes: A nationwide comparative effectiveness study**

**Running head:** Fixed-dose combination drugs and cardiorenal outcomes

**Author names**

Qiaoling Liu ^1,2^, Paul Welsh ^1^, Carlos Celis-Morales ^1,3,4^, Jennifer S. Lees ^1^, Patrick B. Mark^1^, Laura Pazzagli ^2*^

**Affiliations**

^1^School of Cardiovascular and Metabolic Health, University of Glasgow, Glasgow, The United Kingdom

^2^ Clinical Epidemiology Division, Department of Medicine Solna, Karolinska Institutet, Stockholm, Sweden

^3^Human Performance Lab, Education, Physical Activity and Health Research Unit, Universidad Católica del Maule, Talca, Chile

^4^High-Altitude Medicine Research Centre (CEIMA), Universidad Arturo Prat, Iquique, Chile

**Corresponding author**

Laura Pazzagli

Clinical Epidemiology Division

Department of Medicine Solna

Karolinska Institutet

171 77 Stockholm

Sweden

Email: laura.pazzagli@ki.se

**Contents**

eTable 1 Identified antidiabetic SGLT2i/DPP4i/TZD drugs in the study population

eTable 2 Study outcomes and their ICD-10 codes

eTable 3 List of covariates, their definitions, and data source

eTable 4 Paired ATC groups for propensity score matching at ATC code level

eTable 5 Baseline characteristics by drug class of the add-on antidiabetic drug in the unmatched population

eTable 6 Time trend of the proportion of initiators of an LDC and an FDC drug in the unmatched population

eTable 7 Time trend of proportions of antidiabetic drug filled prescriptions by drug class and combination types in the unmatched population

eTable 8 Propensity score matching results of index year, place of residence, and ATC codes

eTable 9 Distribution of outcomes in the unmatched and matched populations

eTable 10 Hazard ratios (HR) following propensity score matching, with pre-matching stratification by drug class

eTable 11 Summary table of sensitivity analysis

eTable 12 Hazard ratios (HR) following propensity score matching on all covariates and diabetes duration

eTable 13 Hazard ratios (HR) following propensity score matching on all covariates in which drug class was replaced by ATC codes

eTable 14 Hazard ratios (HR) for the stratified analyses following propensity score matching on all covariates in which drug class was replaced by ATC codes, for heart failure outcome only

eTable 15 Hazard ratios (HR) following propensity score matching, all outcomes after the index date were used (removing 90-day lag time)

eTable 16 Hazard ratios (HR) following propensity score matching, in population without history of ischaemic heart disease

eTable 17 Hazard ratios (HR) for the stratified analyses following propensity score matching, in population without history of ischaemic heart disease, for heart failure outcome only

eFigure 1 Directed acyclic graph for this study

**eTable 1 Identified antidiabetic SGLT2i/DPP4i/TZD drugs in the study population**

| ATC code | Drug class | Drug name |
| --- | --- | --- |
| A10BD03 | Biguanides+TZD | metformin and rosiglitazone |
| A10BD05 | Biguanides+TZD | metformin and pioglitazone |
| A10BD07 | Biguanides+DPP4i | metformin and sitagliptin |
| A10BD08 | Biguanides+DPP4i | metformin and vildagliptin |
| A10BD10 | Biguanides+DPP4i | metformin and saxagliptin |
| A10BD11 | Biguanides+DPP4i | metformin and linagliptin |
| A10BD15 | Biguanides+SGLT2i | metformin and dapagliflozin |
| A10BD20 | Biguanides+SGLT2i | metformin and empagliflozin |
| A10BD23 | Biguanides+SGLT2i | metformin and ertugliflozin |
| A10BG02 | TZD | rosiglitazone |
| A10BG03 | TZD | pioglitazone |
| A10BH01 | DPP4i | sitagliptin |
| A10BH02 | DPP4i | vildagliptin |
| A10BH03 | DPP4i | saxagliptin |
| A10BH05 | DPP4i | linagliptin |
| A10BK01 | SGLT2i | dapagliflozin |
| A10BK03 | SGLT2i | empagliflozin |
| A10BK04 | SGLT2i | ertugliflozin |

TZD, Thiazolidinedione; DPP4i, Dipeptidyl Peptidase-4 Inhibitor; SGLT2i, Sodium-Glucose Cotransporter 2 Inhibitor; ATC, Anatomical Therapeutic Chemical.

**eTable 2 Study outcomes and their ICD-10 codes**

|  | ICD-10 codes |
| --- | --- |
| Unstable angina | I20.0 |
| Myocardial infarction | I21.* |
| Atrial fibrillation | I48.0~2 |
| Heart failure | I50.* |
| Ischemic stroke | I63.* |

**eTable 3 List of covariates, their definitions, and data source**

|  | Source | Definition |
| --- | --- | --- |
| Age | TPR | Age in years by cohort entry |
| Sex | TPR | Male and female |
| Antidiabetic drug class |  |  |
| SGLT2i | PDR | ATC codes: A10BD15, A10BD20, A10BD23, A10BK01, A10BK03, A10BK04 |
| DPP4i | PDR | ATC codes: A10BD07, A10BD08, A10BD10, A10BD11, A10BH01, A10BH02, A10BH03, A10BH05 |
| TZD | PDR | ATC codes: A10BD03, A10BD05, A10BG02, A10BG03 |
| Comorbidities |  |  |
| Atrial fibrillation | NPR | ICD-10 codes: I48.0~2 |
| Angina | NPR | ICD-10 codes: I20 |
| Atherosclerosis | NPR | ICD-10 codes: I70 |
| Cardiomyopathy | NPR | ICD-10 codes: I42 |
| Heart failure | NPR | ICD-10 codes: I50 |
| Chronic ischemic heart disease | NPR | ICD-10 codes: I25 |
| Myocardial infarction | NPR | ICD-10 codes: I21 |
| Ischemic stroke | NPR | ICD-10 codes: I63 |
| Hemorrhage stroke | NPR | ICD-10 codes: I60~I62 |
| Hypertension | NPR | ICD-10 codes: I10~I13, I15 |
| Acute kidney injury | NPR | ICD-10 codes: N17 |
| CKD Stage 1 | NPR | ICD-10 codes: N18.1 |
| CKD Stage 2 | NPR | ICD-10 codes: N18.2 |
| CKD Stage 3 | NPR | ICD-10 codes: N18.3 |
| Proteinuria | NPR | ICD-10 codes: R80 |
| Diabetic nephrology | NPR | ICD-10 codes: E11.2 |
| Diabetic neuropathy | NPR | ICD-10 codes: E11.4 |
| Diabetic retinopathy | NPR | ICD-10 codes: E11.3 |
| Anemia | NPR | ICD-10 codes: D50~D53, D55~D59 |
| Alcoholic liver disease | NPR | ICD-10 codes: K70 |
| Cirrhosis | NPR | ICD-10 codes: K74.0 |
| Chronic hepatitis | NPR | ICD-10 codes: B18, K73 |
| Hyperthyroidism | NPR | ICD-10 codes: E03, E05 |
| Hypoparathyroidism | NPR | ICD-10 codes: E20, E21 |
| Asthma | NPR | ICD-10 codes: J45 |
| Smoking | NPR | ICD-10 codes: F17, Z72.0 |
| Drinking | NPR | ICD-10 codes: F10 |
| Dyslipidemia | NPR | ICD-10 codes: E78 |
| Obesity | NPR | ICD-10 codes: E66 |
| Recent CVD events^a^ | NPR | ICD-10 codes: I20, I21, I25, I42, I48.0~2, I50, I60~I63, I70 |
| Recent renal events^a^ | NPR | ICD-10 codes: N17, N18.1~3, R80 |
| Medication History |  |  |
| Antihypertensives | PDR | ATC codes: C09 |
| Beta blocker | PDR | ATC codes: C07 |
| Calcium channel blocker | PDR | ATC codes: C08 |
| Diuretics | PDR | ATC codes: C03 |
| Lipid regulators | PDR | ATC codes: C10A |
| Antithrombotics | PDR | ATC codes: B01A |
| Corticosteroids | PDR | ATC codes: H02 |
| NSAIDs | PDR | ATC codes: M01A |
| Marital status | TPR | Unmarried, married, separated, widowed. |
| Place of residence | TPR | 21 counties of Sweden |

^a^Recent event: Event occurred within 1 year prior to the cohort entry.

ATC, Anatomical Therapeutic Chemical; ICD, International Classification of Disease; SGLT2i, sodium-glucose cotransporter 2 inhibitor; DPP4i, dipeptidyl peptidase 4 inhibitor; TZD, thiazolidinedione; CKD, chronic kidney disease; CVD, cardiovascular disease; NSAIDs, non-steroidal anti-inflammatory drugs; NPR, Swedish National Patient Register; PDR, Swedish Prescribed Drug Register; NDR, Swedish National Diabetes Register; TPR, Total Population Register.

**eTable 4 Paired ATC groups for propensity score matching at ATC code level**

| LDC | Paired FDC |
| --- | --- |
| A10BG02 (rosiglitazone) | A10BD03 (metformin and rosiglitazone) |
| A10BG03 (pioglitazone) | A10BD05 (metformin and pioglitazone) |
| A10BH01 (sitagliptin) | A10BD07 (metformin and sitagliptin) |
| A10BH02 (vildagliptin) | A10BD08 (metformin and vildagliptin) |
| A10BH03 (saxagliptin) | A10BD10 (metformin and saxagliptin) |
| A10BH05 (linagliptin) | A10BD11 (metformin and linagliptin) |
| A10BK01 (dapagliflozin) | A10BD15 (metformin and dapagliflozin) |
| A10BK03 (empagliflozin) | A10BD20 (metformin and empagliflozin) |
| A10BK04 (ertugliflozin) | A10BD23 (metformin and ertugliflozin) |

LDC, loose-dose combination; FDC, fixed-dose combination; ATC, Anatomical Therapeutic Chemical.

**eTable 5 Baseline characteristics by drug class of the add-on antidiabetic drug in the unmatched population**

|  | Drug class of the add-on antidiabetic drug | | |
| --- | --- | --- | --- |
|  | SGLT2i | DPP4i | TZD |
| N (row%) | 25,761 (39.4) | 35,728 (54.7) | 3,816 (5.8) |
| Age in years, mean (SD) | 63.0 (11.5) | 63.0 (12.1) | 59.9 (11.2) |
| Male, N (%) | 17,755 (68.9) | 22,285 (62.4) | 2,384 (62.5) |
| Comorbidities, N (%) |  |  |  |
| Atrial fibrillation | 2,156 (8.4) | 2,147 (6.0) | 115 (3.0) |
| Angina | 3,334 (12.9) | 2,947 (8.2) | 215 (5.6) |
| Atherosclerosis | 333 (1.3) | 423 (1.2) | 31 (0.8) |
| Cardiomyopathy | 310 (1.2) | 221 (0.6) | 15 (0.4) |
| Heart failure | 1,375 (5.3) | 1,061 (3.0) | 47 (1.2) |
| Chronic ischemic heart disease | 3,719 (14.4) | 2,404 (6.7) | 124 (3.2) |
| Myocardial infarction | 3,551 (13.8) | 2,320 (6.5) | 121 (3.2) |
| Ischemic stroke | 1,277 (5.0) | 1,502 (4.2) | 74 (1.9) |
| Hemorrhage stroke | 303 (1.2) | 327 (0.9) | 15 (0.4) |
| Hypertension | 2,697 (10.5) | 3,113 (8.7) | 206 (5.4) |
| Acute kidney injury | 94 (0.4) | 150 (0.4) | 5 (0.1) |
| CKD Stage 1 | 16 (0.1) | 12 (0.0) | 0 (0.0) |
| CKD Stage 2 | 27 (0.1) | 25 (0.1) | <5 (<0.1) |
| CKD Stage 3 | 42 (0.1) | 55 (0.2) | 0 (0.0) |
| Proteinuria | 26 (0.1) | 32 (0.1) | 0 (0.0) |
| Diabetic nephrology | 48 (0.2) | 104 (0.3) | 14 (0.4) |
| Diabetic neuropathy | 96 (0.4) | 160 (0.4) | 14 (0.4) |
| Diabetic retinopathy | 1,062 (4.1) | 1,301 (3.6) | 117 (3.1) |
| Anemia | 415 (1.6) | 447 (1.3) | 31 (0.8) |
| Alcoholic liver disease | 51 (0.2) | 69 (0.2) | 5 (0.1) |
| Cirrhosis | 70 (0.3) | 58 (0.2) | <5 (<0.1) |
| Chronic hepatitis | 232 (0.9) | 289 (0.8) | 26 (0.7) |
| Hyperthyroidism | 381 (1.5) | 460 (1.3) | 20 (0.5) |
| Hypoparathyroidism | 131 (0.5) | 182 (0.5) | 11 (0.3) |
| Asthma | 711 (2.8) | 832 (2.3) | 69 (1.8) |
| Smoking | 57 (0.2) | 50 (0.1) | <5 (<0.1) |
| Drinking | 704 (2.7) | 759 (2.1) | 70 (1.8) |
| Dyslipidemia | 419 (1.6) | 353 (1.0) | 19 (0.5) |
| Obesity | 936 (3.6) | 951 (2.7) | 106 (2.8) |
| Recent CVD events, N (%)^a^ | 3,401 (13.2) | 2,237 (6.3) | 145 (3.8) |
| Recent renal events, N (%)^b^ | 75 (0.3) | 138 (0.4) | <5 (<0.1) |
| Drug, N (%) |  |  |  |
| Antihypertensives | 18,929 (73.5) | 23,915 (66.9) | 2,272 (59.5) |
| Beta blocker | 13,283 (51.6) | 15,641 (43.8) | 1,368 (35.8) |
| Calcium channel blocker | 11,227 (43.6) | 13,769 (38.5) | 1,025 (26.9) |
| Diuretics | 9,253 (35.9) | 11,924 (33.4) | 1,067 (28.0) |
| Lipid regulators | 20,290 (78.8) | 25,715 (72.0) | 2,329 (61.0) |
| Antithrombotics | 13,544 (52.6) | 16,118 (45.1) | 1,554 (40.7) |
| Corticosteroids | 7,598 (29.5) | 8,584 (24.0) | 471 (12.3) |
| NSAIDs | 18,330 (71.2) | 23,219 (65.0) | 1,747 (45.8) |

^a^Recent CVD events: Angina, atherosclerosis, atrial fibrillation, cardiomyopathy, chronic ischemic heart disease, heart failure, myocardial infarction, and stroke, occurring within 1 year prior to the cohort entry.

^b^Recent kidney events: Acute kidney injury, CKD Stage 1~3, and proteinuria occurring within 1 year prior to the cohort entry.

SGLT2i, sodium-glucose cotransporter-2 inhibitor; DPP4i, dipeptidyl peptidase-4 inhibitor; TZD, thiazolidinedione; CKD, chronic kidney disease; CVD, cardiovascular disease; NSAIDs, nonsteroidal anti-inflammatory drug; SD, standard deviation; LDC, loose-dose combination; FDC, fixed-dose combination.

Numbers smaller than five were presented as “<5” in accordance with data anonymization standards to minimize the risk of re-identification.

**eTable 6 Time trend of the proportion of initiators of an LDC and an FDC drug in the unmatched population**

|  | LDC | FDC |
| --- | --- | --- |
| Index year, N (row%) |  |  |
| 2006 | 313 (45.5) | 375 (54.5) |
| 2007 | 732 (52.4) | 665 (47.6) |
| 2008 | 1,065 (80.0) | 267 (20.0) |
| 2009 | 906 (68.9) | 409 (31.1) |
| 2010 | 1,032 (75.5) | 336 (24.5) |
| 2011 | 1,202 (77.5) | 349 (22.5) |
| 2012 | 1,328 (79.6) | 341 (20.4) |
| 2013 | 1,647 (80.8) | 392 (19.2) |
| 2014 | 2,045 (78.6) | 558 (21.4) |
| 2015 | 2,904 (80.4) | 708 (19.6) |
| 2016 | 3,752 (80.4) | 919 (19.6) |
| 2017 | 4,914 (80.1) | 1,228 (19.9) |
| 2018 | 6,013 (81.4) | 1,374 (18.6) |
| 2019 | 6,604 (81.1) | 1,540 (18.9) |
| 2020 | 6,688 (78.0) | 1,891 (22.0) |
| 2021 | 7,719 (77.2) | 2,292 (22.8) |
| 2022 | 2,155 (77.1) | 642 (22.9) |

LDC, loose-dose combination; FDC, fixed-dose combination.

**eTable 7 Time trend of proportions of antidiabetic drug filled prescriptions by drug class and combination types in the unmatched population**

|  | Unmatched, LDC group | | | |
| --- | --- | --- | --- | --- |
|  | SGLT2i | DPP4i | TZD | Total |
| N, row (%) | 20,136 (39.5) | 28,537 (55.9) | 2,346 (4.6) | 51,019 (100.0) |
| Year, N (%) |  |  |  |  |
| 2006 | 0 (0.0) | 0 (0.0) | 313 (13.3) | 313 (0.6) |
| 2007 | 0 (0.0) | 238 (0.8) | 494 (21.1) | 732 (1.4) |
| 2008 | 0 (0.0) | 791 (2.8) | 274 (11.7) | 1,065 (2.1) |
| 2009 | 0 (0.0) | 729 (2.6) | 177 (7.5) | 906 (1.8) |
| 2010 | 0 (0.0) | 909 (3.2) | 123 (5.2) | 1,032 (2.0) |
| 2011 | 0 (0.0) | 1,091 (3.8) | 111 (4.7) | 1,202 (2.4) |
| 2012 | 0 (0.0) | 1,278 (4.5) | 50 (2.1) | 1,328 (2.6) |
| 2013 | 63 (0.3) | 1,523 (5.3) | 61 (2.6) | 1,647 (3.2) |
| 2014 | 251 (1.2) | 1,762 (6.2) | 32 (1.4) | 2,045 (4.0) |
| 2015 | 458 (2.3) | 2,407 (8.4) | 39 (1.7) | 2,904 (5.7) |
| 2016 | 767 (3.8) | 2,935 (10.3) | 50 (2.1) | 3,752 (7.4) |
| 2017 | 1,389 (6.9) | 3,428 (12.0) | 97 (4.1) | 4,914 (9.6) |
| 2018 | 2,641 (13.1) | 3,265 (11.4) | 107 (4.6) | 6,013 (11.8) |
| 2019 | 3,328 (16.5) | 3,140 (11.0) | 136 (5.8) | 6,604 (12.9) |
| 2020 | 4,128 (20.5) | 2,449 (8.6) | 111 (4.7) | 6,688 (13.1) |
| 2021 | 5,426 (26.9) | 2,163 (7.6) | 130 (5.5) | 7,719 (15.1) |
| 2022 | 1,685 (8.4) | 429 (1.5) | 41 (1.7) | 2,155 (4.2) |

|  | Unmatched, FDC group | | | |
| --- | --- | --- | --- | --- |
|  | Metformin+SGLT2i | Metformin+DPP4i | Metformin+TZD | Total |
| N, row (%) | 5,625 (39.4) | 7,191 (50.3) | 1,470 (10.3) | 14,286 (100.0) |
| Year, N (%) |  |  |  |  |
| 2006 | 0 (0.0) | 0 (0.0) | 375 (25.5) | 375 (2.6) |
| 2007 | 0 (0.0) | 0 (0.0) | 665 (45.2) | 665 (4.7) |
| 2008 | 0 (0.0) | 50 (0.7) | 217 (14.8) | 267 (1.9) |
| 2009 | 0 (0.0) | 302 (4.2) | 107 (7.3) | 409 (2.9) |
| 2010 | 0 (0.0) | 260 (3.6) | 76 (5.2) | 336 (2.4) |
| 2011 | 0 (0.0) | 342 (4.8) | 7 (0.5) | 349 (2.4) |
| 2012 | 0 (0.0) | 338 (4.7) | <5 (<0.3) | 341 (2.4) |
| 2013 | 0 (0.0) | 389 (5.4) | <5 (<0.3) | 392 (2.7) |
| 2014 | 14 (0.2) | 543 (7.6) | <5 (<0.3) | 558 (3.9) |
| 2015 | 13 (0.2) | 694 (9.7) | <5 (<0.3) | 708 (5.0) |
| 2016 | 136 (2.4) | 780 (10.8) | <5 (<0.3) | 919 (6.4) |
| 2017 | 326 (5.8) | 900 (12.5) | <5 (<0.3) | 1,228 (8.6) |
| 2018 | 611 (10.9) | 762 (10.6) | <5 (<0.3) | 1,374 (9.6) |
| 2019 | 800 (14.2) | 737 (10.2) | <5 (<0.3) | 1,540 (10.8) |
| 2020 | 1,318 (23.4) | 570 (7.9) | <5 (<0.3) | 1,891 (13.2) |
| 2021 | 1,845 (32.8) | 444 (6.2) | <5 (<0.3) | 2,292 (16.0) |
| 2022 | 562 (10.0) | 80 (1.1) | 0 (0.0) | 642 (4.5) |

LDC, loose-dose combination; FDC, fixed-dose combination.

SGLT2i, sodium-glucose cotransporter-2 inhibitor; DPP4i, dipeptidyl peptidase-4 inhibitor; TZD, thiazolidinedione;

Numbers smaller than five were presented as “<5” in accordance with data anonymization standards to minimize the risk of re-identification.

**eTable 8 Propensity score matching results of index year, place of residence, and ATC codes**

**Index year:**

|  | Before matching | | After matching | | Standardized percentage bias |
| --- | --- | --- | --- | --- | --- |
|  | LDC group | FDC group | LDC group | FDC group |  |
| Index year, N (%) |  |  |  |  |  |
| 2006 | 313 (0.6) | 375 (2.6) | 304 (2.2) | 367 (2.6) | 3.6 |
| 2007 | 732 (1.4) | 665 (4.7) | 685 (4.9) | 656 (4.7) | -1.2 |
| 2008 | 1,065 (2.1) | 267 (1.9) | 280 (2.0) | 259 (1.9) | -1.1 |
| 2009 | 906 (1.8) | 409 (2.9) | 501 (3.6) | 406 (2.9) | -4.5 |
| 2010 | 1,032 (2.0) | 336 (2.4) | 362 (2.6) | 326 (2.3) | -1.8 |
| 2011 | 1,202 (2.4) | 349 (2.4) | 408 (2.9) | 340 (2.4) | -3.2 |
| 2012 | 1,328 (2.6) | 341 (2.4) | 394 (2.8) | 333 (2.4) | -2.8 |
| 2013 | 1,647 (3.2) | 392 (2.7) | 426 (3.1) | 369 (2.7) | -2.4 |
| 2014 | 2,045 (4.0) | 558 (3.9) | 578 (4.2) | 538 (3.9) | -1.5 |
| 2015 | 2,904 (5.7) | 708 (5.0) | 727 (5.2) | 677 (4.9) | -1.6 |
| 2016 | 3,752 (7.4) | 919 (6.4) | 925 (6.7) | 878 (6.3) | -1.3 |
| 2017 | 4,914 (9.6) | 1,228 (8.6) | 1,249 (9.0) | 1,179 (8.5) | -1.8 |
| 2018 | 6,013 (11.8) | 1,374 (9.6) | 1,364 (9.8) | 1,320 (9.5) | -1.0 |
| 2019 | 6,604 (12.9) | 154 (10.8) | 1,472 (10.6) | 1,495 (10.8) | 0.5 |
| 2020 | 6,688 (13.1) | 1,891 (13.2) | 1,660 (12.0) | 1,850 (13.3) | 4.0 |
| 2021 | 7,719 (15.1) | 2,292 (16.0) | 2,008 (14.5) | 2,251 (16.2) | 4.8 |
| 2022 | 2,155 (4.2) | 642 (4.5) | 540 (3.9) | 639 (4.6) | 3.5 |

**Places of residence:**

|  | Before matching | | After matching | | Standardized percentage bias |
| --- | --- | --- | --- | --- | --- |
|  | LDC group | FDC group | LDC group | FDC group |  |
| County, N (%) |  |  |  |  |  |
| Stockholm | 7,233 (14.3) | 1,936 (13.9) | 2,005 (14.4) | 1,936 (13.9) | -1.4 |
| Uppsala | 1,723 (3.4) | 349 (2.5) | 364 (2.6) | 349 (2.5) | -0.6 |
| Södermanland | 1,906 (3.8) | 346 (2.5) | 352 (2.5) | 346 (2.5) | -0.2 |
| Östergötland | 1,501 (3.0) | 2,573 (18.5) | 1,498 (10.8) | 2,569 (18.5) | 25.7 |
| Jönköping | 1,854 (3.7) | 896 (6.5) | 1,384 (10.0) | 896 (6.5) | -16.1 |
| Kronoberg | 1,038 (2.1) | 103 (0.7) | 92 (0.7) | 103 (0.7) | 0.7 |
| Kalmar | 1,259 (2.5) | 259 (1.9) | 274 (2.0) | 259 (1.9) | -0.7 |
| Gotland | 340 (0.7) | 44 (0.3) | 46 (0.3) | 44 (0.3) | -0.2 |
| Blekinge | 1,027 (2.0) | 91 (0.7) | 96 (0.7) | 91 (0.7) | -0.3 |
| Skåne | 7,349 (14.5) | 1,517 (10.9) | 1,467 (10.6) | 1,517 (10.9) | 1.1 |
| Hallands | 1,909 (3.8) | 410 (3.0) | 428 (3.1) | 410 (3.0) | -0.7 |
| Västra Götaland | 8,506 (16.8) | 1,167 (8.4) | 1,212 (8.7) | 1,167 (8.4) | -1.0 |
| Värmland | 2,407 (4.8) | 560 (4.0) | 563 (4.1) | 560 (4.0) | -0.1 |
| Örebro | 2,103 (4.2) | 183 (1.3) | 158 (1.1) | 183 (1.3) | 1.1 |
| Västmanland | 1,579 (3.1) | 373 (2.7) | 382 (2.8) | 373 (2.7) | -0.4 |
| Dalarna | 1,954 (3.9) | 469 (3.4) | 494 (3.6) | 469 (3.4) | -1.0 |
| Gävleborg | 2,035 (4.0) | 372 (2.7) | 385 (2.8) | 372 (2.7) | -0.5 |
| Västernorrland | 1,677 (3.3) | 312 (2.2) | 309 (2.2) | 312 (2.2) | 0.1 |
| Jämtland | 779 (1.5) | 259 (1.9) | 332 (2.4) | 259 (1.9) | -4.1 |
| Västerbotten | 117 (2.3) | 1,122 (8.1) | 1,168 (8.4) | 1,122 (8.1) | -1.5 |
| Norrbotten | 1,171 (2.3) | 546 (3.9) | 874 (6.3) | 546 (3.9) | -13.6 |

**ATC codes:**

|  | Before matching | | After matching | | Standardized percentage bias |
| --- | --- | --- | --- | --- | --- |
|  | LDC group | FDC group | LDC group | FDC group |  |
| ATC pairs, N (row %) |  |  |  |  |  |
| Metformin + Dapagliflozin / Dapagliflozin | 3,029 (94.4) | 182 (5.6) | 220 (56.2) | 172 (43.8) | -1.9 |
| Metformin + Emapgliflozin / Empagliflozin | 16,791 (75.7) | 5,402 (24.3) | 4,532 (45.8) | 5,373 (54.2) | 12.6 |
| Metformin + Ertugliflozin / Ertugliflozin | 31 (45.6) | 37 (54.4) | 31 (47.7) | 34 (52.3) | 0.6 |
| Metformin + Sitagliptin / Sitagliptin | 24,876 (78.7) | 6,761 (21.3) | 7,664 (54.1) | 6,501 (45.9) | -16.8 |
| Metformin + Vildagliptin / Vildagliptin | 222 (49.6) | 226 (50.4) | 199 (54.2) | 168 (45.8) | -2.5 |
| Metformin + Saxagliptin / Saxagliptin | 596 (90.9) | 60 (9.1) | 65 (54.6) | 54 (45.4) | -0.9 |
| Metformin + Linagliptin / Linagliptin | 3,091 (95.5) | 148 (4.5) | 251 (63.9) | 142 (36.1) | -4.3 |
| Metformin + Rosiglitazone / Rosiglitazone | 869 (37.9) | 1,424 (62.1) | 855 (38.0) | 1,395 (62.0) | 16.9 |
| Metformin + Pioglitazone / Pioglitazone | 1,514 (97.1) | 46 (2.9) | 66 (60.0) | 44 (40.0) | -1.3 |

LDC, loose-dose combination; FDC, fixed-dose combination; ATC, Anatomical Therapeutic Chemical.

**eTable 9 Distribution of outcomes in the unmatched and matched populations**

|  | Unmatched population | | Matched population | |
| --- | --- | --- | --- | --- |
|  | LDC | FDC | LDC | FDC |
| N (row%) | 51,019 (78.1) | 14,286 (21.9) | 13,883 (50.0) | 13,883 (50.0) |
| Cardiovascular outcomes, N (%) |  |  |  |  |
| Acute MI | 1,451 (2.8) | 459 (3.2) | 485 (3.5) | 449 (3.2) |
| Atrial fibrillation | 1,350 (2.6) | 358 (2.5) | 378 (2.7) | 354 (2.5) |
| Unstable angina | 1,687 (3.3) | 578 (4.0) | 553 (4.0) | 564 (4.1) |
| Heart failure | 2,414 (4.7) | 587 (4.1) | 677 (4.9) | 573 (4.1) |
| Stroke | 1,304 (2.6) | 374 (2.6) | 364 (2.6) | 368 (2.7) |
|  |  |  |  |  |
|  | Unmatched population | | Matched population | |
|  | LDC | FDC | LDC | FDC |
| N (row%) | 51,019 (78.1) | 14,286 (21.9) | 6,864 (50.0) | 6,864 (50.0) |
| Kidney outcome, N (%) |  |  |  |  |
| eGFRcr<30 ml/min/1.73m^2^ | 873 (1.7) | 196 (1.4) | 102 (1.5) | 83 (1.2) |

MI, myocardial infarction; LDC, loose-dose combination; FDC, fixed-dose combination; eGFR, estimated glomerular filtration rate.

**eTable 10 Hazard ratios (HR) following propensity score matching, with pre-matching stratification by drug class**

|  | SGLT2i | DPP4i | TZD |
| --- | --- | --- | --- |
| Sample size, N | 11,150 | 13,725 | 2,866 |
| Cardiovascular outcomes, HR (95%CI) |  |  |  |
| Acute MI | 1.24 (0.90, 1.71) | 1.05 (0.88, 1.25) | 0.94 (0.73, 1.20) |
| Atrial fibrillation | 1.19 (0.89, 1.61) | 1.00 (0.82, 1.21) | 0.88 (0.62, 1.24) |
| Unstable angina | 1.20 (0.80, 1.80) | 0.91 (0.69, 1.19) | 0.84 (0.55, 1.28) |
| Heart failure | 0.94 (0.75, 1.18) | 0.92 (0.78, 1.07) | 0.86 (0.68, 1.09) |
| Ischemic stroke | 0.95 (0.65, 1.38) | 1.02 (0.84, 1.24) | 1.04 (0.80, 1.36) |
|  |  |  |  |
| Sample size, N | 6,150 | 6,844 | 738 |
| Kidney outcome, HR (95%CI) |  |  |  |
| eGFRcr<30 ml/min/1.73m^2^ | 1.73 (0.41, 7.23) | 0.78 (0.55, 1.09) | 1.09 (0.58, 2.06) |

MI, myocardial infarction; LDC, loose-dose combination; FDC, fixed-dose combination; eGFR, estimated glomerular filtration rate; SGLT2i, sodium-glucose cotransporter-2 inhibitor; DPP4i, dipeptidyl peptidase-4 inhibitor; TZD, thiazolidinedione.

**eTable 11 Summary table of sensitivity analyses**

|  | Extra matched on the diabetes duration | Replaced drug class with paired ATC codes | No 90-day lag on outcomes | In population without history of chronic IHD, MI, and unstable angina |
| --- | --- | --- | --- | --- |
| Sample size | 18,250 | 27,766 | 27,766 | 22,979 |
| Cardiovascular outcomes |  |  |  |  |
| Acute MI | 0.93 (0.78, 1.10) | 1.00 (0.88, 1.14) | 0.94 (0.82, 1.06) | 0.96 (0.82, 1.12) |
| Atrial fibrillation | 0.94 (0.77, 1.13) | 1.04 (0.90, 1.21) | 0.95 (0.82, 1.09) | 1.04 (0.88, 1.23) |
| Unstable angina | 0.87 (0.67, 1.12) | 0.88 (0.72, 1.06) | 0.88 (0.73, 1.07) | 0.86 (0.67, 1.11) |
| Heart failure | 0.92 (0.79, 1.07) | 0.91 (0.80, 1.02) | 0.84 (0.75, 0.94) | 0.92 (0.80, 1.05) |
| Ischemic stroke | 0.97 (0.80, 1.17) | 0.99 (0.85, 1.14) | 1.00 (0.86, 1.15) | 0.99 (0.84, 1.17) |
|  |  |  |  |  |
| Sample size | 13,728 | 13,728 | 13,728 | 11,188 |
| Kidney outcome |  |  |  |  |
| eGFRcr<30 ml/min/1.73m^2^ | 0.89 (0.66, 1.19) | 0.84 (0.63, 1.12) | 0.84 (0.63, 1.12) | 0.96 (0.69, 1.33) |

IHD, ischaemic heart disease, MI, myocardial infarction; eGFR, estimated glomerular filtration rate.

**eTable 12 Hazard ratios (HR) following propensity score matching on all covariates and diabetes duration**

|  | HR (95%CI) |
| --- | --- |
| Cardiovascular outcomes |  |
| Acute MI | 0.93 (0.78, 1.10) |
| Atrial fibrillation | 0.94 (0.77, 1.13) |
| Unstable angina | 0.87 (0.67, 1.12) |
| Heart failure | 0.92 (0.79, 1.07) |
| Ischemic stroke | 0.97 (0.80, 1.17) |
|  |  |
| Kidney outcome |  |
| eGFRcr<30 ml/min/1.73m^2^ | 0.89 (0.66, 1.19) |

Sample size for cardiovascular outcomes is 18,250, for kidney outcome, the size is 13,728.

MI, myocardial infarction; eGFR, estimated glomerular filtration rate.

**eTable 13 Hazard ratios (HR) following propensity score matching on all covariates in which drug class was replaced by ATC codes**

|  | HR (95%CI) |
| --- | --- |
| Cardiovascular outcomes |  |
| Acute MI | 1.00 (0.88, 1.14) |
| Atrial fibrillation | 1.04 (0.90, 1.21) |
| Unstable angina | 0.88 (0.72, 1.06) |
| Heart failure | 0.91 (0.80, 1.02) |
| Ischemic stroke | 0.99 (0.85, 1.14) |
|  |  |
| Kidney outcome |  |
| eGFRcr<30 ml/min/1.73m2 | 0.84 (0.63, 1.12) |

Sample size for cardiovascular outcomes is 27,766, for kidney outcome, the size is 13,728.

MI, myocardial infarction; eGFR, estimated glomerular filtration rate.

**eTable 14 Hazard ratios (HR) for the stratified analyses following propensity score matching on all covariates in which drug class was replaced by ATC codes, for heart failure outcome only**

|  | Sample size | HR (95%CI) |
| --- | --- | --- |
| Age |  |  |
| Below 65 years | 15,668 | 1.06 (0.87, 1.28) |
| 65 years and older | 12,098 | 0.82 (0.71, 0.94) |
|  |  |  |
| Sex |  |  |
| Male | 18,591 | 0.91 (0.80, 1.04) |
| Female | 9,175 | 0.90 (0.78, 1.11) |

**eTable 15 Hazard ratios (HR) following propensity score matching, all outcomes after the index date were used (removing 90-day lag time)**

|  | Acute MI | Atrial fibrillation | Unstable angina | Heart failure | Ischemic stroke | eGFRcr  <30 ml/min/1.73m^2^ |
| --- | --- | --- | --- | --- | --- | --- |
| Events during follow-up, n | 954 | 746 | 416 | 1,283 | 757 | 193 |
| Follow-up time in years, Median (IQR) | 4.0 (1.9, 7.2) | 4.0 (1.9, 7.3) | 4.0 (1.9, 7.4) | 4.0 (1.9, 7.3) | 4.0 (1.9, 7.3) | 3.8 (1.9, 6.3) |
| Incident rate per 1000 person-years, (95%CI) | 6.6 (6.2, 7.1) | 5.1 (4.8, 5.5) | 2.8 (2.6, 3.1) | 8.9 (8.4, 9.4) | 5.2 (4.9, 5.6) | 3.0 (2.6, 3.4) |
|  |  |  |  |  |  |  |
| Hazard Ratio (95%CI) | 0.94 (0.82, 1.06) | 0.95 (0.82, 1.09) | 0.88 (0.73, 1.07) | 0.84 (0.75, 0.94) | 1.00 (0.86, 1.15) | 0.84 (0.63, 1.12) |

Sample size for cardiovascular outcomes is 27,766, for kidney outcome, the size is 13,728.

MI, myocardial infarction; eGFR, estimated glomerular filtration rate.

**eTable 16 Hazard ratios (HR) following propensity score matching, in population without history of ischaemic heart disease**

|  | HR (95%CI) |
| --- | --- |
| Cardiovascular outcomes |  |
| Acute MI | 0.96 (0.82, 1.12) |
| Atrial fibrillation | 1.04 (0.88, 1.23) |
| Unstable angina | 0.86 (0.67, 1.11) |
| Heart failure | 0.92 (0.80, 1.05) |
| Ischemic stroke | 0.99 (0.84, 1.17) |
|  |  |
| Kidney outcome |  |
| eGFRcr<30 ml/min/1.73m^2^ | 0.96 (0.69, 1.33) |

Sample size for cardiovascular outcomes is 22,979, for kidney outcome, the size is 11,188.

MI, myocardial infarction; eGFR, estimated glomerular filtration rate.

**eTable 17 Hazard ratios (HR) for the stratified analyses following propensity score matching, in population without history of ischaemic heart disease, for heart failure outcome only**

|  | Sample size | HR (95%CI) |
| --- | --- | --- |
| Age |  |  |
| Below 65 years | 13,982 | 1.01 (0.88, 1.37) |
| 65 years and older | 8,997 | 0.78 (0.66, 0.93) |
|  |  |  |
| Sex |  |  |
| Male | 14,812 | 0.91 (0.77, 1.08) |
| Female | 8,167 | 0.93 (0.73, 1.18) |

**eFigure 1 Directed acyclic graph for this study**


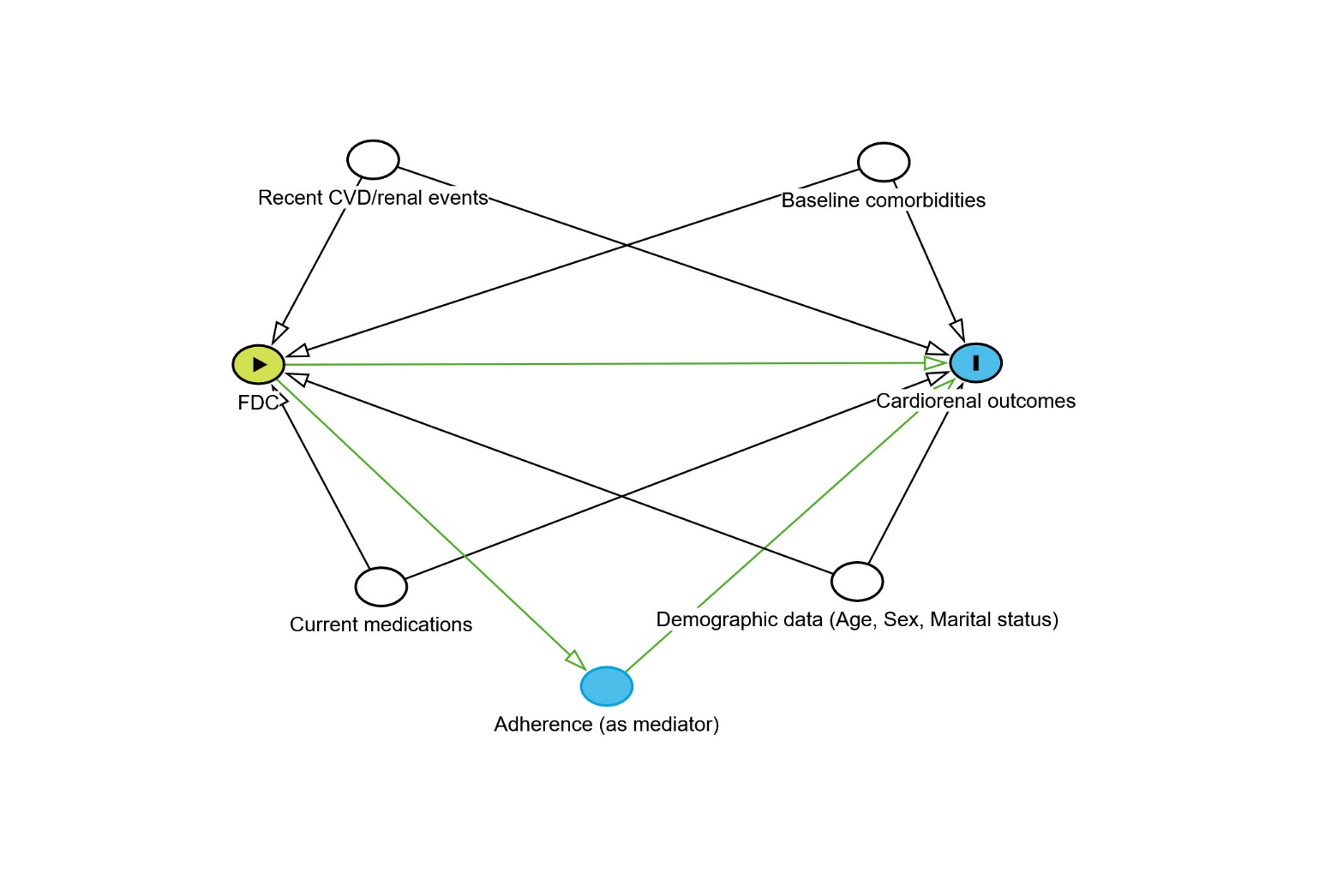


Solid green circle, exposure; solid blue circle without character “I”, mediator; solid blue circle with character “I”, outcome; solid blank circle, adjusted confounding factors; solid black line, confounding path; solid green line, causal path of interest; arrow, direction.
